# Supplementary material for: Rapid active zone remodeling consolidates presynaptic potentiation
Source: Nat Commun. 2019 Mar 6;10:1085. doi: 10.1038/s41467-019-08977-6 (PMC6403334; doi:10.1038/s41467-019-08977-6)
Supplement: Supplementary file 2 — Description of Additional Supplementary Files [file 41467_2019_8977_MOESM2_ESM.pdf]

Description of Additional Supplementary Files

File Name: Supplementary Movie 1
Description: Axonal Co-transport of BRP and Unc13A. Live imaging in intact third instar larvae of axonal (segment A2) BRP (green) and Unc13A (magenta) showed anterograde co-transport of both proteins.
